# A large genomic deletion leads to enhancer adoption by the lamin B1 gene: a second path to autosomal dominant adult-onset demyelinating leukodystrophy (ADLD)

**DOI:** 10.1093/hmg/ddv065

**Published:** 2015-02-20

**Authors:** Elisa Giorgio, Daniel Robyr, Malte Spielmann, Enza Ferrero, Eleonora Di Gregorio, Daniele Imperiale, Giovanna Vaula, Georgios Stamoulis, Federico Santoni, Cristiana Atzori, Laura Gasparini, Denise Ferrera, Claudio Canale, Michel Guipponi, Len A. Pennacchio, Stylianos E. Antonarakis, Alessandro Brussino, Alfredo Brusco

**Affiliations:** 1 Department of Medical Sciences, University of Torino, via Santena, 19, Torino 10126, Italy; 2 Department of Genetic Medicine and Development, University of Geneva Medical School, Geneva 1211, Switzerland; 3 Max Planck Institute for Molecular Genetics, Ihnestr. 63-73, Berlin 14195, Germany; 4 Medical Genetics Unit and; 5 Department of Neurology, Città della Salute e della Scienza University Hospital, Torino 10126, Italy; 6 Centro Regionale Malattie Da Prioni - Domp (ASLTO2), Torino 10144, Italy; 7 Department of Neuroscience and Brain Technologies and; 8 Department of Nanophysics, Istituto Italiano di Tecnologia, Genoa 16163, Italy and; 9 Genomics Division, Lawrence Berkeley National Laboratory, MS 84-171, Berkeley, CA 9472, USA

## Abstract

Chromosomal rearrangements with duplication of the lamin B1 (*LMNB1*) gene underlie autosomal dominant adult-onset demyelinating leukodystrophy (ADLD), a rare neurological disorder in which overexpression of *LMNB1* causes progressive central nervous system demyelination. However, we previously reported an ADLD family (ADLD-1-TO) without evidence of duplication or other mutation in *LMNB1* despite linkage to the *LMNB1* locus and lamin B1 overexpression. By custom array-CGH, we further investigated this family and report here that patients carry a large (∼660 kb) heterozygous deletion that begins 66 kb upstream of the *LMNB1* promoter. Lamin B1 overexpression was confirmed in further ADLD-1-TO tissues and in a postmortem brain sample, where lamin B1 was increased in the frontal lobe. Through parallel studies, we investigated both loss of genetic material and chromosomal rearrangement as possible causes of *LMNB1* overexpression, and found that ADLD-1-TO plausibly results from an enhancer adoption mechanism. The deletion eliminates a genome topological domain boundary, allowing normally forbidden interactions between at least three forebrain-directed enhancers and the *LMNB1* promoter, in line with the observed mainly cerebral localization of lamin B1 overexpression and myelin degeneration. This second route to *LMNB1* overexpression and ADLD is a new example of the relevance of regulatory landscape modifications in determining Mendelian phenotypes.

## Introduction

Autosomal dominant adult-onset demyelinating leukodystrophy (ADLD, OMIM #169 500) is a rare neurological disorder characterized by genetically determined, progressive loss of white matter (WM) within the central nervous system (CNS) (1). Most forms of leukodystrophy are early onset, appearing in childhood. In contrast, the clinical onset of ADLD occurs in the fourth or fifth decade, usually with symptoms of autonomic dysfunction, followed by ataxia and cognitive impairment that signal pyramidal and cerebellar involvement. Typical changes associated with demyelination are observed in brain and spinal cord WM by MRI (2).

The familial occurrence of ADLD has been reported in linkage with chromosome 5q23-31 in a dozen families of broadly different ethnic origins (2–10), including one Italian family that we described in 2010 (11). Genetic analyses ultimately led to the identification by Padiath *et al*. of heterozygous duplications of the lamin B1 gene (*LMNB1*, chr5q23.2) as the disease-causing mutations in ADLD (9). Thus the disease is caused by increased levels of lamin B1 protein produced by the presence of a functional extra copy of the *LMNB1* gene, making ADLD as part of a growing number of neurological disorders caused by gene copy number variation.

The B-type lamins are members of the intermediate filament protein superfamily and play a major role in forming the nuclear lamina lining the inner nuclear membrane. Lamin B1 appears to play two distinct functions in the vertebrate nucleus: a structural role in maintaining nuclear integrity (12–14), and a regulatory role in DNA replication and gene expression (15,16). Overexpression of lamin B1 in human (HEK 293) and murine (NG2a) cell lines has been reported to increase nuclear rigidity, a feature also present in nuclei obtained from ADLD skin fibroblasts (14).

Although lamin B1 is ubiquitously expressed it appears that oligodendrocytes, the cells responsible for myelin deposition in the CNS, are particularly sensitive to *LMNB1* gene dosage. In these cells, lamin B1 overexpression perturbs both nuclear architecture and gene expression, leading to demyelination through a complex pathway that involves downregulation of proteolipid protein (PLP, a major myelin sheath component whose duplication leads to Pelizaeus-Merzbacher Disease, OMIM#300401) by reduced Yin-Yang 1 transcription factor (TF) binding (17,18).

Since 2010, we have been studying a multigenerational Italian family, designated ADLD-1-TO, in which affected members present clinical and neuroradiological signs compatible with ADLD (19). However, subtle differences were noted, such as the absence of autonomic dysfunction at onset and relative sparing of cerebellar WM (11,20). By linkage analysis, we mapped the disease to chromosome 5q23.2-q23.3, within a 4.3 Mb genomic region containing *LMNB1*. We demonstrated that lamin B1 mRNA expression in ADLD-1-TO patients was increased and comparable to the expression levels found in ADLD patients with *LMNB1* duplication (9,21,22). However, we found neither duplication, mutation nor deletion in *LMNB1* (11).

This paper reports on our efforts to reach a genetic diagnosis in the ALDL-1-TO family.

## Results

### Increased expression of LMNB1 in ADLD-1-TO tissues

**Figure 1.**
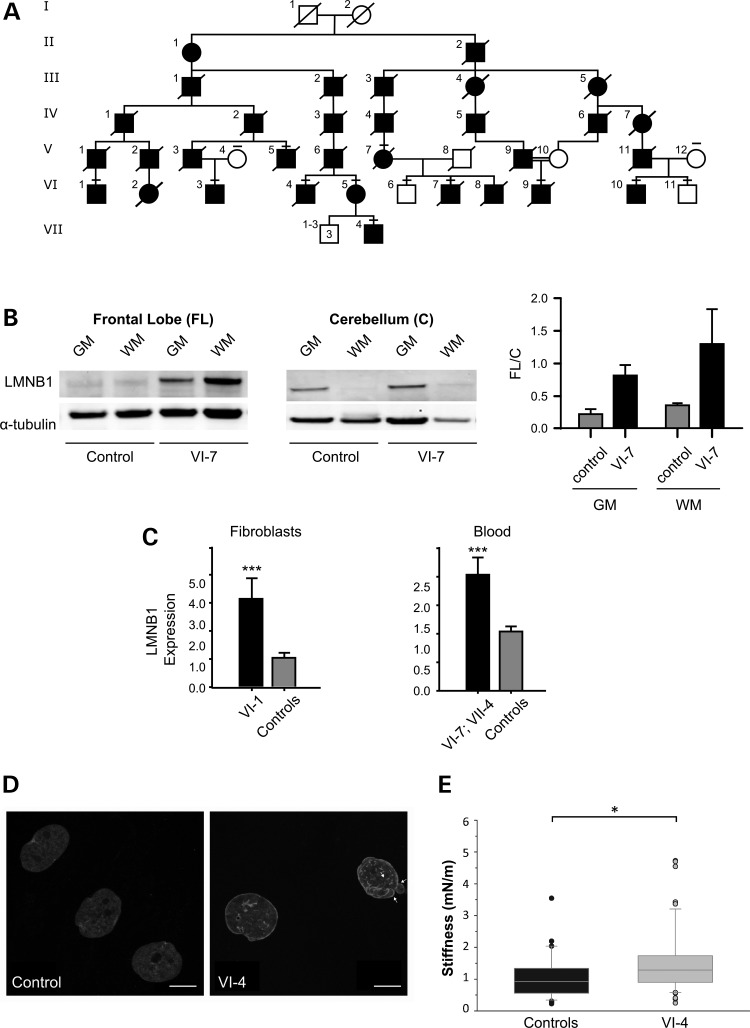
ADLD-1-TO family, *LMNB1* expression and nuclear abnormalities. (**A**) Simplified ADLD-1-TO family tree. Most of the healthy subjects have been omitted. A short line over the patient symbol indicates DNA was available for the study. (**B**) Western blot analysis of LMNB1 from FL (left) and cerebellum (center) GM and WM. Twenty micrograms of brain protein extracts from a control and an ADLD-1-TO (VI-7) patient were analyzed. The histogram (right) shows LMNB1 protein levels detected by western blot as a ratio between FL and cerebellum (C) (loading control alpha-tubulin); our patient presented an FL/C ratio in GM of 0.81 versus 0.22 in the control, and in WM of 1.36 versus 0.36. (**C**) Real-time PCR Lamin B1 (*LMNB1*) levels measured in RNA derived from fibroblasts or PAXgene-stabilized blood. Both experiments are normalized versus the *HMBS* gene and the value on the y-axis represents the dose calculated with the 2^−ΔΔCt^ method. Error bars indicates Standard Error. ***P < 0.001, Mann–Whitney two-tailed test. (**D**) Representative maximal projections of z-stack confocal images of nuclei from control and ADLD-1-TO human skin fibroblasts (VI-4) immunostained for LMNB1. Scale bar: 10 µm. (**E**) Nuclear stiffness analysis by AFM was performed on nuclei extracted from quiescent control and ADLD-1-TO fibroblasts (VI-4). Box plot shows nuclear stiffness values. A total of 88 fibroblast nuclei from three age-matched control subjects (*n* = 34 nuclei) and one ADLD-1-TO (*n* = 54 nuclei) patient were analyzed in three independent experiments. *P < 0.05, Mann–Whitney Rank Sum Test.

Overexpression of lamin B1 is a hallmark of ADLD (9,19,22) but the most disease-relevant tissue, the brain, is also the least accessible. We obtained a postmortem brain specimen following autopsy of patient VI-7 (Fig. 1A) who died at the age of 56 due to ADLD. Western blot analysis of a frontal lobe (FL) extract showed a robust increase in lamin B1 protein (about 7-fold) with respect to a control postmortem brain specimen (Fig. 1B). Bearing in mind that MRI in ADLD-1-TO patients shows that forebrain-derived structures are affected, but not the cerebellum (hindbrain-derived) (19,21), we analyzed LMNB1 protein levels also in the cerebellum. Comparison between FL and cerebellar (C) LMNB1 protein levels by western blot demonstrated that in the patient, lamin B1 is about 3.6 times more abundant in FL compared to the control [FL/C ratio of 0.81 versus 0.22 in gray matter (GM), and 1.23 versus 0.36 in WM, Fig. 1B].

Lamin B1 overexpression was also detected in proxy tissues from patients: by real-time polymerase chain reaction (PCR), lamin B1 transcripts were increased 2- to 3-fold in fibroblasts from patient VI-1 (Fig. 1C; mean ± standard error = 3.15 ± 0.73; *P* < 0.0001) and in PAXgene-stabilized whole blood obtained from patients VI-7 and VII-4 (Fig. 1C, mean ± standard error = 2.04 ± 0.31; *P* < 0.0001) with respect to healthy controls.

### Altered physical properties of nuclei from ADLD-1-TO cells

In ADLD with *LMNB1* duplication, the surplus lamin B1 protein is found mostly in the nuclear lamina, leading to alterations in nuclear morphology and increased nuclear membrane rigidity (14,17). In fibroblasts from patient VI-1, we observed: (i) lamin B1 protein accumulation within the nuclear lamina by immunocytochemistry (Fig. 1D); (ii) abnormal nuclear morphology with blebs and invaginations by fluorescence microscopy (Fig. 1D) and (iii) ∼44% increase in nuclear rigidity compared to control nuclei by atomic force microscopy (AFM) indentation (Fig. 1E; *P* < 0.05), with a significant increase of Young's elastic modulus (average ± SEM: 707.0 ± 159.1 Pa) compared to control nuclei (421.9 ± 60.6 Pa; *P* = 0.02, Student's *t*-test. Data not shown).

### Identification of a large deletion 66 kb upstream of LMNB1in ADLD-1-TO

**Figure 2.**
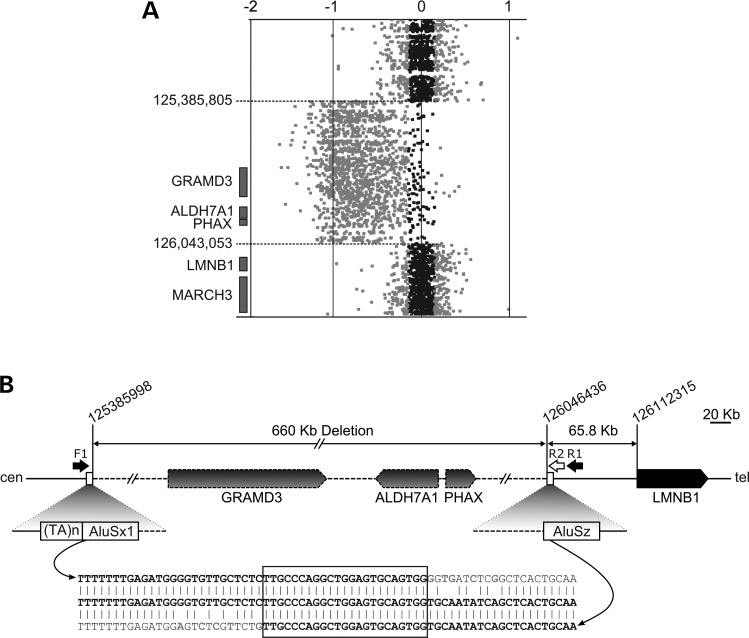
Characterization of a large deletion 66 kb upstream of *LMNB1*. (**A**) Custom a-CGH graphical output. Values on top represent the log ratio of the probes (log_2_ intensity of Cy5/Cy3 fluorochromes): expected values are from −0.7 to −1 for a deletion, 0 (zero) for normal and 0.5–1 for a genomic duplication. The position of the first normal probe is reported on the left, along with a schematic representation of the genes involved (gray rectangles). (**B**) Schematic of the deleted genomic region in which the deleted genes have dashed border and the arrowhead shows the transcription direction. The position of the primers used to amplify and sequence the breakpoint (not in scale) is shown (black and white arrows; F1, R1 and R2). The two Alu elements (AluSx1 and AluSz) and a (TA)_n_ repeat are shown. Below, the sequence of the breakpoint: a stretch of 23 identical bases are shared between the two regions (boxed).

As the experimental evidence indicated that *LMNB1* overexpression occurred in ADLD-1-TO in the absence of *LMNB1* mutations, we searched for disruptions that might cause aberrant *LMNB1* transcriptional control. Custom array CGH analysis of a 2.2 Mb region of chromosome 5 centered on *LMNB1* identified a 660 kb deletion (hg19/chr5:125 385 805–126 043 053), with the closest deletion boundary located 66 kb upstream of *LMNB1* (Fig. 2A). The deletion was confirmed by FISH analysis (Supplementary Material, Fig. S1). This deletion was not present in the Database of Genomic Variants (http://dgv.tcag.ca/dgv/app/home), and not detected in 100 Italian healthy subjects.

Nucleotide sequences of the deletion boundaries contained *Alu* elements: *AluSx1* at the centromeric boundary and *AluSz* at the telomeric boundary (Fig. 2B). These elements share a 23-bp region of microhomology, suggesting Alu-derived microhomology-mediated break-induced replication (MMBIR) was responsible for the rearrangement (23,24).

The chromosomal rearrangement upstream of *LMNB1* led to the deletion of three genes (Fig. 2B), the closest to *LMNB1* being PHosphorylated Adaptor for RNA eXport (*PHAX,* OMIM *604924), which encodes a 394 amino acid protein involved in snRNA export from the nucleus. The second deleted gene is ALdehyde DeHydrogenase 7 family, member A1 (*ALDH7A1*, OMIM *107323) which encodes an enzyme involved in lysine metabolism; recessive mutations cause pyridoxine-dependent epilepsy (OMIM #266100). The most distal deleted gene is GRAM domain containing 3 (*GRAMD3*) which encodes a broadly expressed 432 amino acid protein found in cytoplasmic microtubules. Although not associated with any neuropathology to our knowledge, we note that it is expressed in oligodendrocyte precursors (www.genecards.org).

### Analysis of the deleted genes effect on LMNB1 expression

*GRAMD3*, *ALDH7A1* and *PHAX* appear to be broadly expressed in humans, including in adult brain (http://www.genecards.org/). Although there is no evidence of a pathway linking these genes to *LMNB1* regulation, we cannot completely exclude that they play a role in ADLD or in lamin B1 gain of expression. For the current study, we established a cellular model that mimicked the effects of the deletion by reducing the amount of *GRAMD3*, *ALDH7A1* and *PHAX* transcripts. We then observed the effects on lamin B1 expression. We generated simultaneous triple-gene haploinsufficiency in normal human fibroblasts by small interfering RNA (siRNA) transfection and measured lamin B1 transcripts. Although mRNA levels of *GRAMD3*, *ALDH7A1* and *PHAX* were reduced by at least 50%, and therefore comparable to those expected in our patient-derived fibroblasts, *LMNB1* expression was unchanged (Supplementary Material, Fig. S2).

### Normal and patient-specific long-range interactions with the LMNB1 promoter

**Figure 3.**
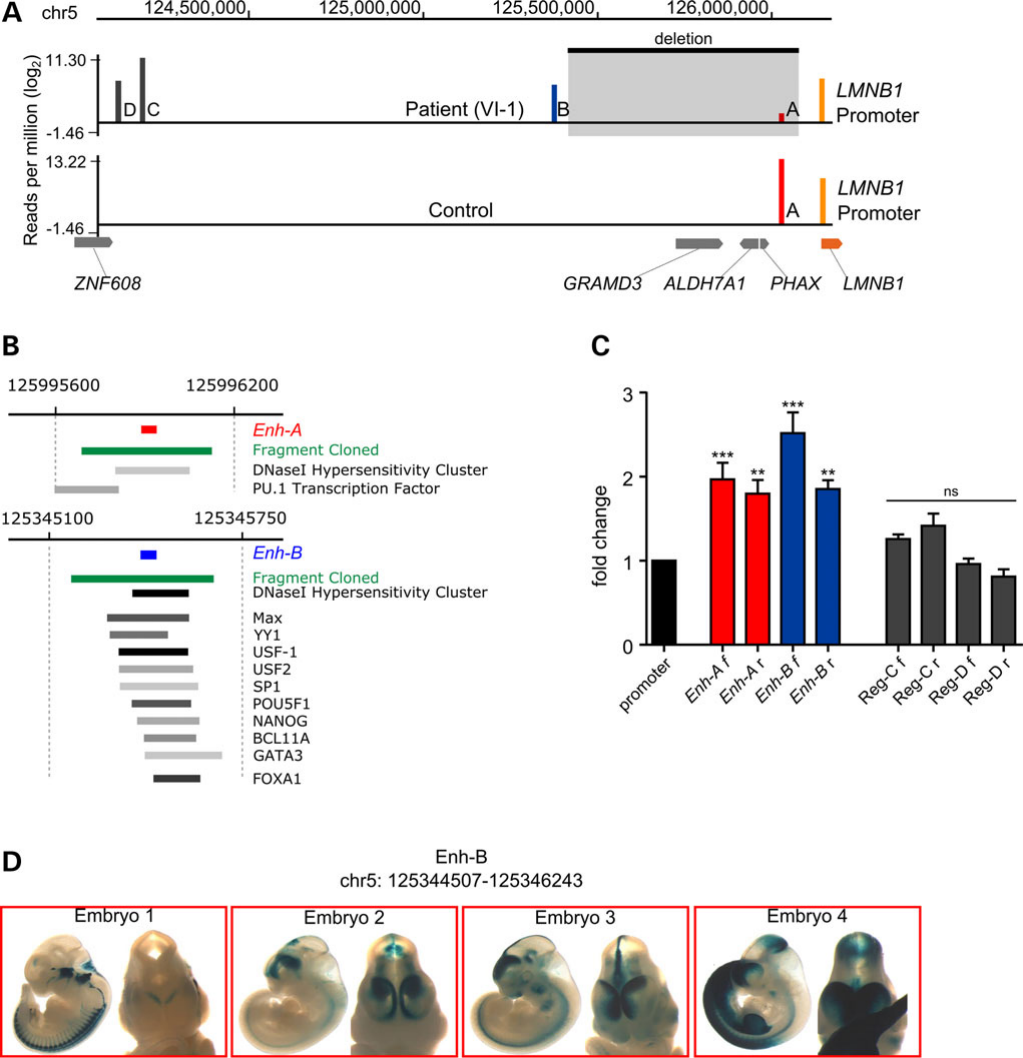
Circular chromosome conformation capture (4C) and *in vitro*/*in vivo* enhancer study. (**A**) Schematic of the 4C results. Summary of the log_2_ interactions (reads/million) with the lamin B1 promoter (orange bars) in patient VI-1 and a control (vertical bars A–D). The deletion is shown as a black rectangle, with gray arrows representing the involved genes and *LMNB1* in orange. (**B**) Integrated Regulation track (modified from UCSC browser) for regions A and B (ENCODE data). Red and blue bars represent regions A and B captured by 4C (DpnII fragments), respectively. Green bars represent enhancer-containing regions cloned to perform *in vitro* validation. Gray bars represent consensus sequences for TFs; bar color intensity is proportional to the level of TF enrichment from the UCSC Uniform TFBS Track. (**C**) Dual luciferase assay on HEK293T cells showing the effect of regions A–D on the *LMNB1* promoter. The luciferase activity is normalized on the pGL4.10 construct containing the *LMNB1* promoter alone (fold change on the y-bar) (see Supplementary Material for a summary of the vectors used). Red and blue bars show the activity of regions A and B in forward (f) and reverse (r) orientation. Dark gray bars show the activity of regions C and D, in forward (f) and reverse (r) orientation. Error bars represent the standard error of the mean (n.s = not significant; *** *P*-value < 0.001, **P-value < 0.01; Mann–Whitney two-tailed test). (**D**) Transgenic mouse enhancer assay with *Enh-B* suggests forebrain-specific enhancer activity at day E11.5.

To identify interactions between the rearranged upstream genomic region and the *LMNB1* promoter that might determine the gain of lamin B1 expression, we performed circular chromosome conformation capture (4C) analyses in fibroblasts of ADLD-1-TO patient VI-1 and a healthy control, using *LMNB1* promoter as bait. This technique was developed to identify physical interactions genome-wide from any given genomic location (25,26). We identified four genomic regions interacting with the *LMNB1* promoter: regions A, B, C and D (Fig. 3A, Supplementary Material, Fig. S3 and 4). Region A was identified in both patient and control, and mapped to ∼120 kb 5′ of the *LMNB1* promoter on the wild type allele. Region A is deleted in the mutant chromosome, but one copy remains available for interaction in the patient. The other three interacting regions were identified only in the patient: regions B, C and D mapped to 0.77, 1.94 and 2.0 Mb upstream of the *LMNB1* promoter respectively (Fig. 3A). As a consequence of the deletion, the three regions are relocated closer to the *LMNB1* promoter in the patient.

### Analysis and validation of 4C contacts

We analyzed regions A, B, C and D by *in silico* prediction of enhancer activity using the ENCODE tracks at UCSC (www.genome.ucsc.edu), and identified putative enhancer features in regions A and B, but not in regions C and D. Region A shows digital DNaseI hypersensitive sites and a potential site for PU1 TF binding; region B has DNaseI hypersensitive sites and putative binding motifs for BCL11A, FOXA1, GATA3, Max, NANOG, POU5F1, SP1, USF-1, USF-2 and Yin Yang 1 TFs (Fig. 3B). None of these regions is evolutionarily conserved.

To test the predicted enhancers experimentally, we performed dual-luciferase reporter assays with constructs containing regions A, B, C or D, in direct or reverse orientation, upstream of the *LMNB1* promoter (for promoter definition, see Supplementary Material and Fig. S5). Following transfection of the constructs into HEK293T human cell line, regions A and B (in both orientations) significantly increased luciferase expression (A forward = 1.97 ± 0.2; A reverse = 1.8 ± 1.6; B forward = 2.52 ± 0.25; B reverse = 1.85 ± 0.10; mean ± standard error; *P* < 0.01; Fig. 3C). In contrast, constructs containing regions C and D did not significantly affect luciferase expression (Fig. 3C). Similar results were obtained when the same plasmids were transfected into NIH3T3 cell line (*data not shown*), suggesting that the enhancer activity of regions A and B could function across species, at least in mouse fibroblasts. Following these results, regions A and B were subsequently referred to as enhancer region A (*Enh-A*) and enhancer region B (*Enh-B*).

To validate and better characterize *Enh-A* and *-B* interactions with the *LMNB1* promoter, we performed nested-PCR on circularized DNA which confirmed that region A interacted in both patient and control, whereas the interaction of region B was patient-specific (Supplementary Material). In addition, for region B we established that the 4C interaction occurred only in *cis* by exploiting a heterozygous SNP in the *LMNB1* promoter of the patient in which the ‘C’ allele segregated with the deletion (Supplementary Material and Fig. S6). Overall, the 4C assay together with the *in silico* and *in vitro* data suggest that the deletion establishes an enhancer-adoption mechanism (27,28) by monoallelic *Enh-A* deletion followed by repositioning of *Enh-B* from ∼770 kb to ∼110 kb upstream of the *LMNB1* promoter.

To determine if *Enh-A* and *Enh-B* behave as enhancers in an *in vivo* murine model, we performed transgenic mouse enhancer assays (29) with *Enh-A* or *Enh-B* linked to a minimal mouse promoter and *lacZ* reporter gene (*Hsp68-lacZ*). Founder transgenic embryos were generated and stained for *lacZ* reporter activity at embryonic day 11.5. Of 17 F_0_ embryos with the *Enh-B* construct, four showed reporter expression in the developing forebrain (Fig. 3D). In contrast, no F_0_ embryos with the *Enh*-A showed reporter expression (data not shown).

### Genomic landscape of LMNB1 locus with topological domains (TDs)

**Figure 4.**
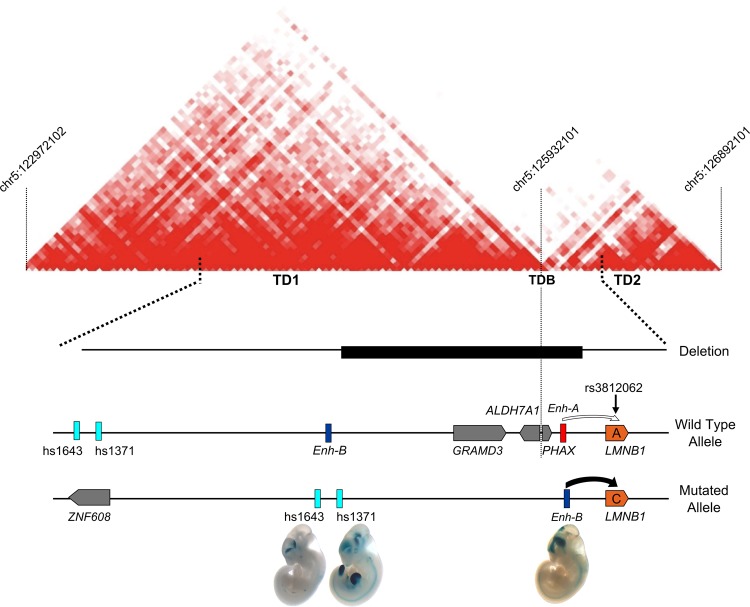
*LMNB1* regulatory landscape. The heat map shows the topological domains (TD1 and TD2) and the regulatory boundary (TDB) between *ALDH7A1* and *PHAX* genes. In wild-type alleles, *Enh-A* interacts with the *LMNB1* promoter. The ∼660 kb deletion removes *Enh*-A and a TDB, likely causing ‘enhancer adoption’. In this situation, three elements may act on the *LMNB1* promoter: *Enh-B,* identified by our 4C experiments, and two human elements, hs1643 and hs1371, annotated as enhancers in the VISTA enhancer browser. All have a forebrain or forebrain/midbrain enhancer activity as shown by *in vivo* mouse enhancer assay.

To validate the hypothesis that the deletion in ADLD-1-TO patients leads to enhancer adoption, we used the genome-wide chromatin interaction data obtained by the Hi-C protocol generated by Dixon *et al.* (30) to analyze the genomic landscape of the 1.5 Mb region surrounding *LMNB1* (http://yuelab.org/hi-c/). This procedure identified both TDs (sub-Mb chromosomal regions that favor enhancer-promoter interactions) and a topological domain boundary (TD boundary or insulator) (31) (Fig. 4) involving *LMNB1*. The first TD (TD1, chr5: 122 972 101–125 932 102) includes *Enh*-B and the *ZNF608*, *GRAMD3* and *ALDH7A1* genes; the second TD (TD2, chr5: 125 932 101–126 852 102) encompasses *PHAX*, *Enh-A* and *LMNB1*. The deletion spans across TD1 and TD2, eliminating the TD boundary element located at chr5: 125 932 101–125 932 102, likely allowing the interaction between *Enh-B* and the *LMNB1* promoter.

## Discussion

Gene duplication, a fortuitous event caused by errors in DNA replication/recombination, represents a major positive force in genome evolution (32). However, gene duplication can also have negative consequences, and give rise to a Mendelian disorder such as autosomal dominant leukodystrophy. The evidence to date, accumulated from affected families of varied ethnicity, has pointed to duplication of the lamin B1 gene on chromosome 5 as the cause of ADLD. Although the presence of excess gene product probably occurs in all cells throughout the body, it is in the brain that excess lamin B1 protein does most damage by mechanisms that are currently being worked out.

We previously described a large Italian family with clinical hallmarks compatible with ADLD and indeed, the disease segregated with the *LMNB1* locus and affected individuals showed increased expression of lamin B1 mRNA and protein. Detailed genetic analysis of the ADLD-1-TO family was negative, as no alterations in *LMNB1* structure or copy number could be found (11). However, when we unexpectedly gained access to postmortem brain specimens from an ADLD-1-TO patient who succumbed to the disease, western blot analyses unequivocally showed dramatically increased amounts of lamin B1. To our knowledge, this is the first time the disease target organ is analyzed in this way. Further support came from the recently published observations by Ferrera *et al.* regarding alterations in the mechanical properties of the nucleus induced by lamin B1 overexpression. In fact, fibroblast-derived nuclei from ADLD-1-TO patients also showed changes in nuclear membrane rigidity and bleb formation overlapping with those reported in *LMNB1*-duplicated ADLD patients (14). The question remained: what was causing lamin B1 overexpression in the enigmatic ADLD-1-TO family?

As increased expression caused by transcriptional dysregulation could obviously mimic gene duplication (33), we again analyzed our family's DNA, this time by array-CGH (a-CGH), finally identifying a 660 kb deletion upstream of *LMNB1*. Besides rearranging this region of chromosome 5, the deletion led to the monoallelic loss of three genes -*GRAMD3*, *ALDH7A1* and *PHAX*. Thus, we needed to address both the effects of (i) relocating formerly distant DNA closer to *LMNB1* and (ii) monoallelic three-gene loss on lamin B1 gene production.

Little to nothing is known of the function of the three deleted genes in CNS myelination, but disease connections for them or related genes have been described. The most distant gene from *LMNB1* is *GRAMD3*, a protein of unknown function containing a GRAM domain (for Glucosyltransferases, Rab-like GTPase activators and Myotubularins). We note that other proteins containing GRAM domains, myotubularins, are altered in Charcot-Marie-Tooth type 4B demyelinating neuropathy (34). *ALDH7A1*, encoding an acetaldehyde-oxidizing enzyme, is mutated in autosomal recessive pyridoxine-dependent epilepsy (OMIM#266100) (35). *PHAX*, encoding a protein identified in mouse as a component of the U snRNA export complex assembly, has recently been associated with the Pierre Robin Sequence (36,37). Although we cannot in principle rule out that these three genes play a role in ADLD-1-TO pathogenesis, it has to be noted that their experimentally induced simultaneous single-copy loss did not affect lamin B1 expression. In addition, we recently described an ADLD patient with duplication not only of *LMNB1* but also of *GRAMD3*, *ALDH7A1* and *PHAX* [patient BR1 in (22)]. In this patient, the increase in lamin B1 expression was comparable to that found in patients with *LMNB1*-only duplication, further suggesting that copy number variation in *GRAMD3*, *ALDH7A1* and *PHAX* does not interfere with *LMNB1* expression. Therefore our favored hypothesis is that these genes act as innocent bystanders with respect to disease pathogenesis. Such a ‘bystander effect’ has been described, for example, in aniridia (OMIM 106210): although a downstream gene, *ELP4*, is deleted by genomic translocations in different patients, it is not the direct cause of the disease which is instead caused by loss of a *PAX6* enhancer (38).

The 4C technique was chosen to search for *LMNB1* regulatory regions relocated in ADLD-1-TO patients following the large deletion. Chromosome Conformation Capture related techniques have already been successfully applied to uncover interactions between distant regulatory elements, as occurs with *CDKN2B* in pancreatic cancer susceptibility or *FOXL2* gene in blepharophimosis syndrome (39–41). Although 4C identified four enhancer-containing regions, only regions A and B were retained; regions C and D had no enhancer-like elements according to ENCODE and were negative by luciferase assay, and therefore excluded from further analysis.

*In silico* analyses support a regulatory function for *Enh-A* and *Enh-B*, even if not evolutionarily conserved. This finding is in accordance with recent papers demonstrating that a large proportion of functional enhancers is not subject to evolutionary constraints (42,43). The ENCODE database shows that both enhancer elements are within DNaseI hypersensitive sites, a marker of open chromatin structure. In addition, several binding sites for TFs are predicted for *Enh-B*: (i) the YY1 and Sp1 TFs, which are active during oligodendrocyte differentiation (44,45) and (ii) BCL11A and GATA3, which are involved in neuron morphogenesis and survival of sympathetic neurons (46,47). It is an intriguing possibility that in ADLD-1-TO *LMNB1* might be placed under the regulation of glial and neuron-specific enhancers. It was also recently suggested that lamin B1 might be a regulator of PLP1 myelin protein through interaction with YY1 TF (18). It seems reasonable to hypothesize the formation of a positive feedback loop of *Enh-B* with its YY1 binding site on *LMNB1*, which may further increase *LMNB1* expression in specific cell types (e.g. glia in the CNS) or developmental stages. Enhancers A and B were validated by nested-PCR on circularized DNA, dual luciferase assay and *in vivo* transgenic mouse experiments.

Recent advances into the demarcation of genomes into functional TDs (30) have shed further light on the consequences of the ALDL-1-TO deletion and rearrangement. Analysis of the TDs surrounding *LMNB1* shows that the deletion eliminates a boundary element that normally separates *Enh-B* from the *LMNB1* promoter. It is likely that disruption of this boundary element can lead to enhancer adoption, i.e. following mutation, a gene acquires a non-physiological enhancer that drives ectopic expression of the gene (31). This mechanism of TD boundary disruption, or TDBD, opens the *LMNB1* promoter to interaction with *Enh-B* and two VISTA enhancer regions located in TD1: (i) human element hs1371, a forebrain-, midbrain- and limb-specific enhancer and (ii) hs1643, a forebrain-specific enhancer (48). Overall, these data suggest that the chromosomal rearrangement caused by the deletion alters the regulatory landscape of *LMNB1*, in turn leading to gene overexpression and disease.

Consistent with the pattern of activity of *Enh-B*, hs1371 and hs1643, MR imaging in ADLD-1-TO patients showed that forebrain-derived structures are affected, but the cerebellum (hindbrain-derived) is spared, a telling difference with ADLD families with *LMNB1* duplication, where WM throughout the brain degenerates (20,21).

In a patient's brain, we found a greater increase in lamin B1 protein in the FL compared to the cerebellum (about 3.6 fold both in gray and in WM); these data, however, are limited by the availability of a single sample and by the advanced stage of the disease, whose effect on lamin B1 levels are poorly known, and need to be confirmed if further brain samples become available. Taken together, these results are consistent with the proposed pathogenic mechanism and the demyelinating lesions in the ADLD-1-TO kindred.

Position effects, notably enhancer adoption and enhancer loss, are among an emerging group of disease-causing mechanisms. Examples of enhancer adoption are Liebenberg syndrome (MIM 186550), a homeotic arm-to-leg transformation, in which deletion and rearrangement brings two active enhancers closer to the *PITX1* (paired-like homeodomain 1) promoter (28,31) or holoprosencephaly spectrum disorder and severe upper limb syndactyly with lower limb synpolydactyly due to the relocation of the *SHH* transcription unit near a limb bud enhancer (49). Instead enhancer loss occurs in Léri-Weill syndrome and BPES (Blepharophimosis, ptosis, epicanthus inversus syndrome), in which deletions of *SHOX* (Léri-Weill) or *FOXL2* (BPES) enhancers are reported (50,51). A recent study demonstrated that in up to 11% of all deletions reported in the DECIPHER database of chromosomal imbalances, disease phenotypes can partly be explained by enhancer adoption (25). Mutations in regulatory regions may be highly underestimated, considering the ENCODE estimate that at least 20% of the human genome, which represents about seven times the amount of DNA coding for exons (2.9%), is involved in regulatory functions (52).

In conclusion, we describe an ADLD family in which disease pathogenesis is caused by overexpression of the lamin B1 gene in the brain. However, in this family ADLD is not caused by the ‘classical’ molecular mechanism of *LMNB1* duplication, but by a deletion upstream of *LMNB1* that destroys a TD boundary and brings heterologous forebrain-specific enhancers to act upon the *LMNB1* promoter. Genomic alterations that change the regulatory landscape are emerging determinants of Mendelian phenotypes and, as our experience shows, need to be taken into account when screening for mutations in these diseases.

## Materials and Methods

### Patients

A simplified pedigree of ADLD-1-TO family is reported in Figure 1A. Signs, symptoms and MRI findings have been described elsewhere (11). Genomic DNA was already available from different family members (black dash above the symbol in Fig. 1A). For this work, we obtained: (i) PAXgene-stabilized blood samples (Qiagen, Mannheim, Germany) from two patients (VI-7; VII-4); (ii) fibroblasts from a skin biopsy of patient VI-1; (iii) autopsy samples from patient VI-7. Control hippocampal tissue was obtained at autopsy from six patients with no history of seizures or other neurological diseases. All autopsies were performed within 12 h after death. The study was approved by the DSM-ChUB Internal Review Board. Informed consent was obtained for the use of blood and skin samples, brain tissue and for the access to medical records for research purposes. Tissue was obtained and used in compliance with the Declaration of Helsinki.

### Array comparative genomic hybridization and breakpoint identification

Two patients, VI-1 and VI-3 (Fig. 1A), were tested for deletion/duplication by custom a-CGH assay designed using the Agilent eArray tool on an 8 × 15 K support (https://earray.chem.agilent.com/earray/, Agilent Technologies, Santa Clara, California, USA). We selected a ∼2.2 Mb region between positions 125 010 000 and 127 269 000 on chromosome 5 (assembly GRCH37/hg19) with an average resolution of one probe every ∼170 bp. Slides were scanned on a G2565BA scanner and analyzed using Agilent CGH Analytics software ver. 4.0.81 (Agilent Technologies).

Array-CGH data were validated using FISH analysis on LCLs metaphase preparations from patient VI-3, using BAC probes RP11–1123C14 and RP11–1031D8 spanning the deleted region, and RP11–322L12 and RP11–638F8 overlapping the centromeric and telomeric breakpoint, respectively (Supplementary Material, Fig. S1). Real-time PCR was also performed to evaluate *GRAMD3* gene copy number under standard conditions (exon 2, primers: 5′-ggtgtggaggagaaaaagaaagc; 5′-ggagtccgcctccacagat; TaqMan probe: 6-FAM-tgcaggtcgccaaca-Quencher). The same real-time PCR was used to screen 100 Italian healthy controls to exclude the deletion was a rare local polymorphism.

Deletion breakpoints were identified by PCR-amplification with primers F1 and R1 (Supplementary Material, Table S1), using the LA Taq PCR Kit (Takara Bio Inc., Otsu, Shiga 520–2193, Japan) in a final volume of 25 µl under the following conditions: 60 ng genomic DNA, 1× Buffer, 0.4 mm dNTPs, 2.5 mm MgCl_2_, 0.5 µM of each primer and 1.25 U of LA Taq polymerase. Cycling conditions were: 94°C for 1 min, 30 cycles at 98°C for 10 s, 62°C for 10 s and 68°C for 6 min, and a final extension at 72°C for 10 min. Polymerase chain reaction was followed by Sanger sequencing with primer R2 on an ABI-Prism 3100 Avant automatic sequencer (Applied Biosystems, Foster City, CA, USA).

### RNA interference experiments

A pool of commercially available siRNAs specific for *GRAMD3* (NM_001146319, siRNA assays number s35303), *ALDH7A1* (NM_001201377.1, s1770) and *PHAX* (NM_032177.3, s28674; Life Technologies, Carlsbad, CA, USA) (Supplementary Material, Table S1 and Fig. S2) were used at final concentrations ranging from 28 to 34 nM to transfect 2.5 × 10^4^ human control primary fibroblasts with the Lipofectamine 2000 reagent, according to manufacturer's instructions (Life Technologies). A scramble siRNA was used as negative control (AM 4620, Life Technologies). The experiment was performed twice with two technical replicates. After 24 h incubation, total RNA was extracted and retrotranscribed with the ‘TaqMan Cells-to-CT’ kit (Life Technologies). Effective silencing was evaluated by Real-Time PCR using commercially available TaqMan assays for *GRAMD3* (Hs01597460_m1), *ALDH7A1* (Hs00609622_m1) and *PHAX* (Hs00536084_m1; Life Technologies) with *HMBS* gene as normalizer (Hs00609297_m1). An expression reduction of at least 50% for each gene was considered optimal. Lamin B1 cDNA level (*LMNB1*, Hs01059210_m1, Life Technologies) was measured by real-time PCR using *HMBS* as normalizer gene (5).

### Western blot analysis

Ten percent (wt/vol) brain homogenates from FL and cerebellum GM and WM of a deceased patient (VI-7, Fig. 1A) and a control subject were prepared in 0.5% deoxycholic acid/sodium deoxycholate with a protease inhibitor cocktail (Roche Diagnostics, Mannheim, Germany). Two different samplings for each region were performed in patient and control with great care to obtain specimens from similar locations. Total protein concentration was assessed by BCA assay (Pierce BCA Protein assay kit, Thermo Scientific, Rockford, IL, USA). Twenty micrograms of protein samples were separated by SDS-polyacrylamide gel electrophoresis (SDS-PAGE) in a 4–12% polyacrylamide gel (Invitrogen, Life Technologies, Carlsbad, CA, USA). Lamin B1 was detected using a primary rabbit anti-lamin B1antibody (AB16048, dilution 1:6000, Abcam) and an enhanced chemiluminescence system (Pierce ECL plus, Thermo Scientific). Rabbit monoclonal anti-alpha tubulin antibody was used as loading control (clone EP1332Y, cat. 04–1117, dilution 1:500, Merck Millipore, Darmstadt, Germany). Images were acquired and analyzed using a VersaDoc Imaging system and the Quantity One software (Bio-Rad, Milan, Italy).

### Immunofluorescence

Fibroblasts were plated onto poly-L-lysine-coated coverslips, serum-deprived for 24 h, fixed in 4% PFA and immunolabeled as previously described (14). The samples were immunostained using rabbit polyclonal anti-LB1 (Abcam) or monoclonal anti-LB1 (Zymed) and counterstained with Hoechst-33342. The confocal optical sectioning was performed at room temperature (RT) using a Leica TCS SP5 AOBS TANDEM inverted confocal microscope that was equipped with a 40 × HCX PL APO 1.25 oil objective lens.

Atomic force microscopy analysis. For the AFM studies, the fibroblasts were analyzed following serum deprivation for 24 h. To avoid confounding effects of cytoskeletal components and force dissipation by cytosolic factors, we performed AFM force spectroscopy on isolated nuclei that were extracted by osmotic lysis of cells with 0.56% KCl for 30 min at RT and spun at 350 × g for 5 min at RT. The nuclei were then re-suspended in PBS and plated onto a poly-L-lysine-coated petri dish by incubation for 1 h at RT. AFM in force spectroscopy mode (53–55) was performed using the Nanowizard II AFM (JPK Instruments, Germany) that was mounted on an Axio Observer D1 inverted optical microscope (Carl Zeiss, Germany). Nuclear elasticity was probed using spherical polystyrene beads (Ø 4 μm; Polysciences Inc., USA) that were mounted on silicon tipless cantilevers TL1 (Nanosensors, Switzerland) with nominal spring constant of 0.03 N/m. The actual spring constant of each cantilever was determined using the *in situ* thermal noise method (56). The maximum force applied to the sample was 1 nN. The velocity of the piezo-scanner was maintained at a constant 3 μm/s. The force curves were corrected for the bending of the cantilever (57) to calculate the tip-sample separation and to build force versus indentation (F–I) curves (Supplementary Material, Fig. S1). Nuclear stiffness was computed as described (58) by calculating the linear fit of the F–I curve between two specific force values (i.e. *F*_o_ = 250 pN and *F*_1_ = 550 pN), corresponding to indentations of ≤1 μm. The force curves were analyzed using a custom MathLab routine to allow the batch processing of a set of curves according to the following formula: *stiffness (mN/m)* = *-* $\left(Y_{1}-Y_{0}/X_{1}-X_{0}\right)$, where *X*_1_ and *X*_0_ are the tip-sample separation in µm, and *Y*_1_ and *Y*_0_ are the deflection in nN at forces of *F*_1_ and F_0_, respectively. A total of 570 nuclei were analyzed. Eight-by-eight curve point force spectroscopy maps (64 curves/nucleus) covering an area of 16 μm^2^ were acquired in the center of each nucleus using the DirectOverlay routine of the AFM acquisition software (JPK Instruments, Germany). Stiffness values between 0.05 and 10 mN/m were accepted (59). Young's elastic modulus (E) was calculated as described (60).

### Statistical analysis

The statistical analysis between the groups with normal distributions was performed using Student's *t*-test for two groups. When the normality test failed, the analysis was performed using non-parametric tests, such as the Mann–Whitney Rank Sum Test. The differences between groups were considered to be statistically significant when *P* < 0.05.

### Circular chromosome conformation capture (4C) assay

Circular Chromosome Conformation Capture was used to search for interactors of the lamin B1 promoter. The only cells available were fibroblasts from a single patient (VI-1). Even if the pathology involves brain, this cell type was considered appropriate for 4C assay given the overexpression of *LMNB1* in this patient's tissue (Fig. 1B). A promoter region between two DpnII restriction sites (chr5:126 112 186–126 112 384; assembly GRCh37/hg19) was selected as a bait. 4C was performed as described elsewhere (36) with the following modifications: fibroblasts from subject VI-1 (Fig. 1A) and an healthy sex-matched control were grown in DMEM supplemented with 10% heat inactivated fetal bovine serum and 100 µg/ml streptomycin/penicillin to obtain a total of ∼1 × 10^7^ cells. Cross-linking with formaldehyde (1% v/v) was performed for 10 min at RT directly in the cell media prior to quenching with 125 mM glycine. Cells were washed in ice-cold PBS, trypsinized for 20 min at 37°C, and washed twice in ice-cold PBS. Cells were lysed for 15 min on ice in 500 µl 10 mM Tris–HCl pH 8.0, 10 mM NaCl, 0.2% v/v IGEPAL CA-630 supplemented with protease inhibitor (Roche Diagnostics). The cell lysate was homogenized with 10 + 10 strokes in a Douncer (‘A’ or tight pestle) and subsequently transferred in a 1.5 ml tube. Cells were pelleted for 5 min (4600 G at 4°C), washed twice in 500 µl of 1× DpnII restriction buffer and re-suspended in 600 µl 1.2× DpnII restriction buffer. Restriction with DpnII, ligation, crosslink reversal and DNA purification were carried out as described previously (37).

The 4C library was generated from 100 ng of ligated DNA with two successive rounds of PCR amplification using two nested pairs of primers (Supplementary Material, Table S1). The PCR conditions were as follows. First amplification: 20 pmol primers and 1× Phusion HF mix (New England BioLabs), thermal cycling conditions were 98°C for 30 s, 35 cycles of 98°C for 10 s, 55°C for 30 s, 72°C for 90 s and a final elongation at 72°C for 3 min. Second amplification: a 1:100 dilution of the first PCR product was amplified with 40 pmoles of nested primers at the following thermal cycling conditions: 94°C for 3 min, 33 cycles of 94°C for 30 s, 65°C for 30 s, 72°C for 90 s and followed by a final elongation at 72°C for 3 min. Primers used during the second round of amplification have additional nucleotides at their 5′ end (Supplementary Material, Table S1) that are required for DNA colony amplification on the cluster station as part of the Illumina Genome Analyzer high-throughput sequencing procedure. The library was gel purified to reduce the amount of DNA originating from self-ligation of the DpnII restricted bait. Sequencing was carried out using an Illumina Genome Analyzer. The sequencing primers were designed to anneal just upstream to the DpnII (GATC) restriction site on one side of the bait. Hence all sequences begin with GATC.

The sequences were aligned against the repeat-masked human genome (build hg18) using the BLAT tool (38). The quality of the alignments was filtered according to the following criteria: (i) the minimum match length was set to 29 nucleotides with no gap larger than one nucleotide; (ii) alignments had to start with the DpnII site as the first nucleotides. All sequences that did not fit these criteria or that aligned to more than one location on the genome were discarded.

Validation of 4C interactors were performed by nested-PCR on circularized DNA (see Supplementary Material).

### Validation of regulatory elements *in vitro*: luciferase assay

Based on the Illumina results, we selected five fragments of ∼450 bp centered on the short read sequence. Fragments were PCR-amplified under standard conditions with primers containing a 5′-KpnI restriction site (Supplementary Material, Table S1). Polymerase chain reaction products were gel purified with the HiYield™ Gel/PCR Fragments Extraction Kit (RBC Bioscience), and inserted into a pTZ57R/T plasmid using the InsTA Cloning PCR Kit (Invitrogen). Plasmids were transformed using the HIT DH5A cells (RBC), and extracted using the PureYield Plasmid Miniprep System (Promega). pTZ57R/T plasmids were digested with KpnI restriction endonuclease, the insert gel-purified and ligated into the pGL4.10 plasmid with the *LMNB1* promoter (long form, see Supplementary Material). Plasmids were transformed into JM109 competent cells (Promega) and extracted using the PureYield Plasmid Midiprep System (Promega). HEK293T human cells and NH3T3 murine fibroblasts were transiently co-transfected with a total of 300 ng of pGL4.10 constructs/pGL4.74 (ratio 30:1), and luminescence measured as described above. For each assay, three independent experiments were performed in duplicate.

### Validation of regulatory elements *in vivo*: transgenic mouse enhancer assay

Enhancer region-B (*Enh-B*; chr5: 125 344 507–125 346 243/hg19) and -A (*Enh-A*; chr5: 125 995 897 125 996 243/hg19) were amplified from human genomic DNA by PCR, sequence-validated and transferred into an Hsp68-LacZ reporter vector. Generation of transgenic mice and embryo staining was done as previously described (29) in accordance with protocols approved by the Lawrence Berkeley National Laboratory. A total of 12 and 17 independent transgenic embryos were obtained, respectively, for regions A and B, and embryos exhibiting LacZ-staining were scored and annotated independently by multiple curators. To be defined as a positive enhancer, an element has to show reproducible expression in the same structure in at least three independent transgenic embryos, each representing an independent oocyte injection with random genomic integration.

### Genomic landscape of LMNB1 locus with TDs

Topological domain data from genome-wide higher-order chromatin interaction data in human embryonic stem cells were downloaded (http://yuelab.org/hi-c/) and mapped to hg19 coordinates using the UCSC liftover tool (30,61). Topological domain boundaries (TDBs) are defined as regions of up to 400 kb between TD regions. We analyzed specifically the 1.5 Mb region surrounding the *LMNB1* gene, including the fragment deleted in the ADLD-1-TO family.

### Bioinformatics web resources

The presence of DNaseI hypersensitive regions, TF binding sites, and histone modification variants was evaluated with the UCSC genome browser (www.genome.ucsc.edu) and using the Integrated Regulation track based on data derived from the ENCODE project. 4C sequences were aligned against the repeat-masked human genome (build hg18) using the BLAT tool (62). VISTA Enhancer browser (enhancer.lbl.gov/) was used to analyze known enhancer regions.

## Supplementary Material

Supplementary Material is available at *HMG* online.

## Funding

This work was supported by Telethon grant number GGP10184 and ELA Foundation grant number 2001-006C2 to A. Brusco and L. Gasparini; ERC grant number 249968 and SNF grant number 144082 to S.E.A. L.A.P. was supported by grants HG003988 and U54HG006997 funded by National Human Genome Research Institute. Part of this research was conducted at the E.O. Lawrence Berkeley National Laboratory and performed under Department of Energy Contract DE-AC02-05CH11231, University of California. Funding to pay the Open Access publication charges for this article was provided by Fondazione Telethon.

## Supplementary Material

Supplementary Data
