# Supplementary material for: A large genomic deletion leads to enhancer adoption by the lamin B1 gene: a second path to autosomal dominant adult-onset demyelinating leukodystrophy (ADLD)
Source: Hum Mol Genet. 2015 Feb 20;24(11):3143–54. doi: 10.1093/hmg/ddv065 (PMC4424952; doi:10.1093/hmg/ddv065)
Supplement: Supplementary Data [file supp_24_11_3143__index.html]

A large genomic deletion leads to enhancer adoption by the lamin B1 gene: a second path to autosomal dominant leukodystrophy (ADLD) — A large genomic deletion leads to enhancer adoption by the lamin B1 gene: a second path to autosomal dominant adult-onset demyelinating leukodystrophy (ADLD) — A large genomic deletion leads to enhancer adoption by the lamin B1 gene: a second path to autosomal dominant adult-onset demyelinating leukodystrophy (ADLD) — Supplementary Data 

# A large genomic deletion leads to enhancer adoption by the lamin B1 gene: a second path to autosomal dominant adult-onset demyelinating leukodystrophy (ADLD)

## Supplementary Data

Supplementary Data

**Files in this Data Supplement:**

- Supplementary Figure 1 - png file
- Supplementary Figure 2 - png file
- Supplementary Figure 3 - png file
- Supplementary Figure 4 - png file
- Supplementary Figure 5 - png file
- Supplementary Figure 6 - png file
