# Supplementary figures and images for: A large genomic deletion leads to enhancer adoption by the lamin B1 gene: a second path to autosomal dominant adult-onset demyelinating leukodystrophy (ADLD)
Source: Hum Mol Genet. 2015 Feb 20;24(11):3143–54. doi: 10.1093/hmg/ddv065 (PMC4424952; doi:10.1093/hmg/ddv065)

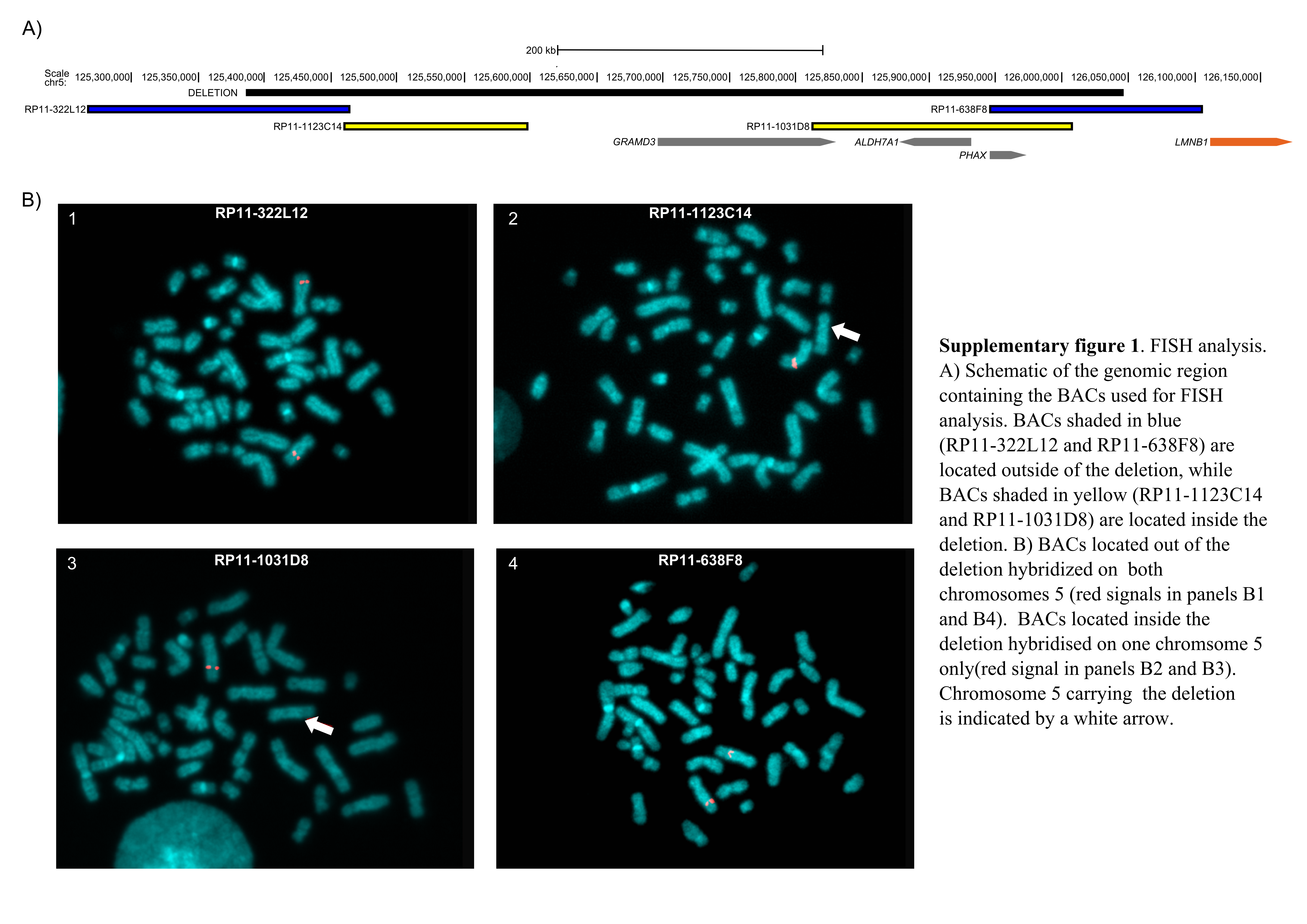

Supplement: Supplementary Data [file supp_ddv065_ddv065supp_fig1.png]

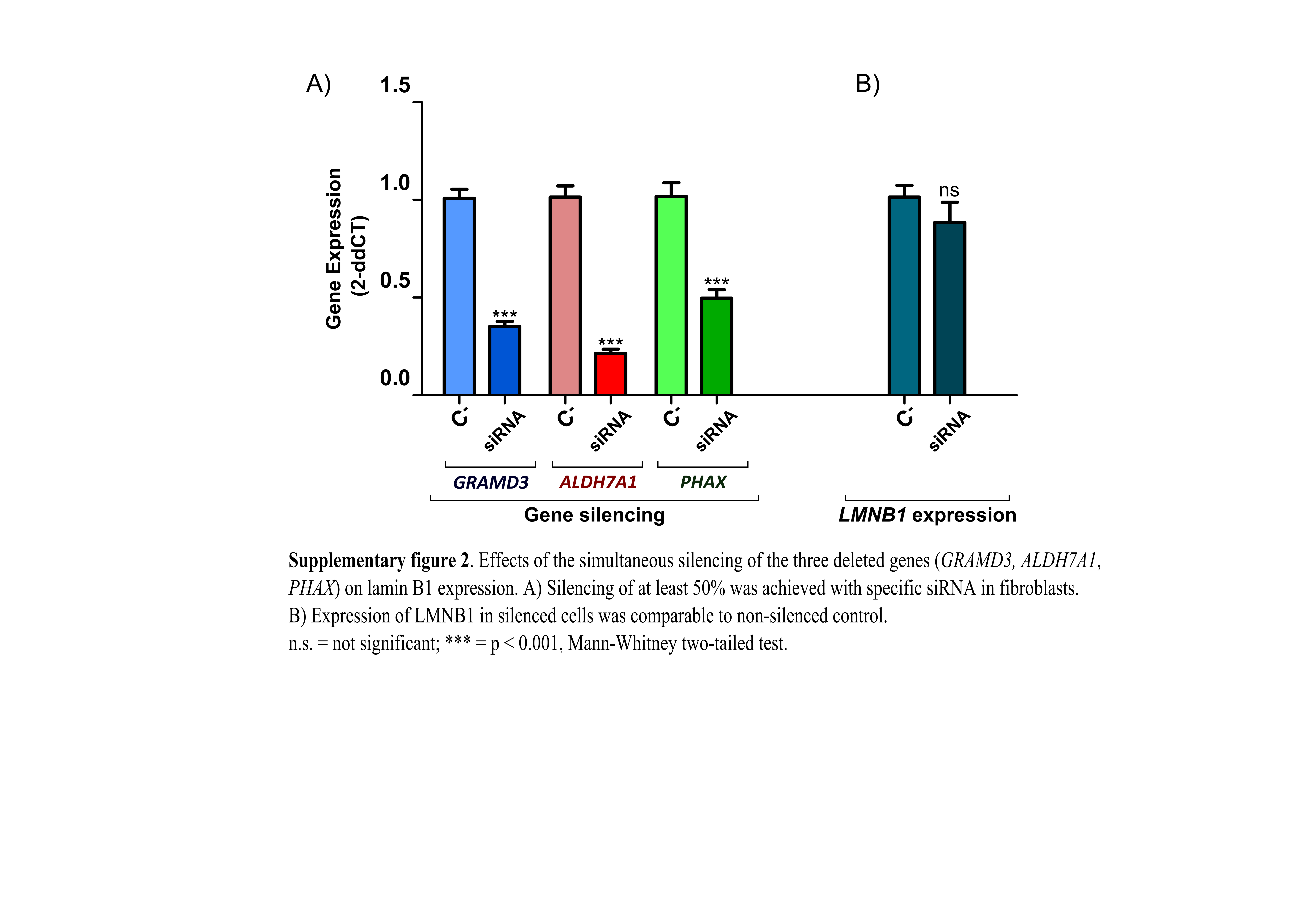

Supplement: Supplementary Data [file supp_ddv065_ddv065supp_fig2.png]

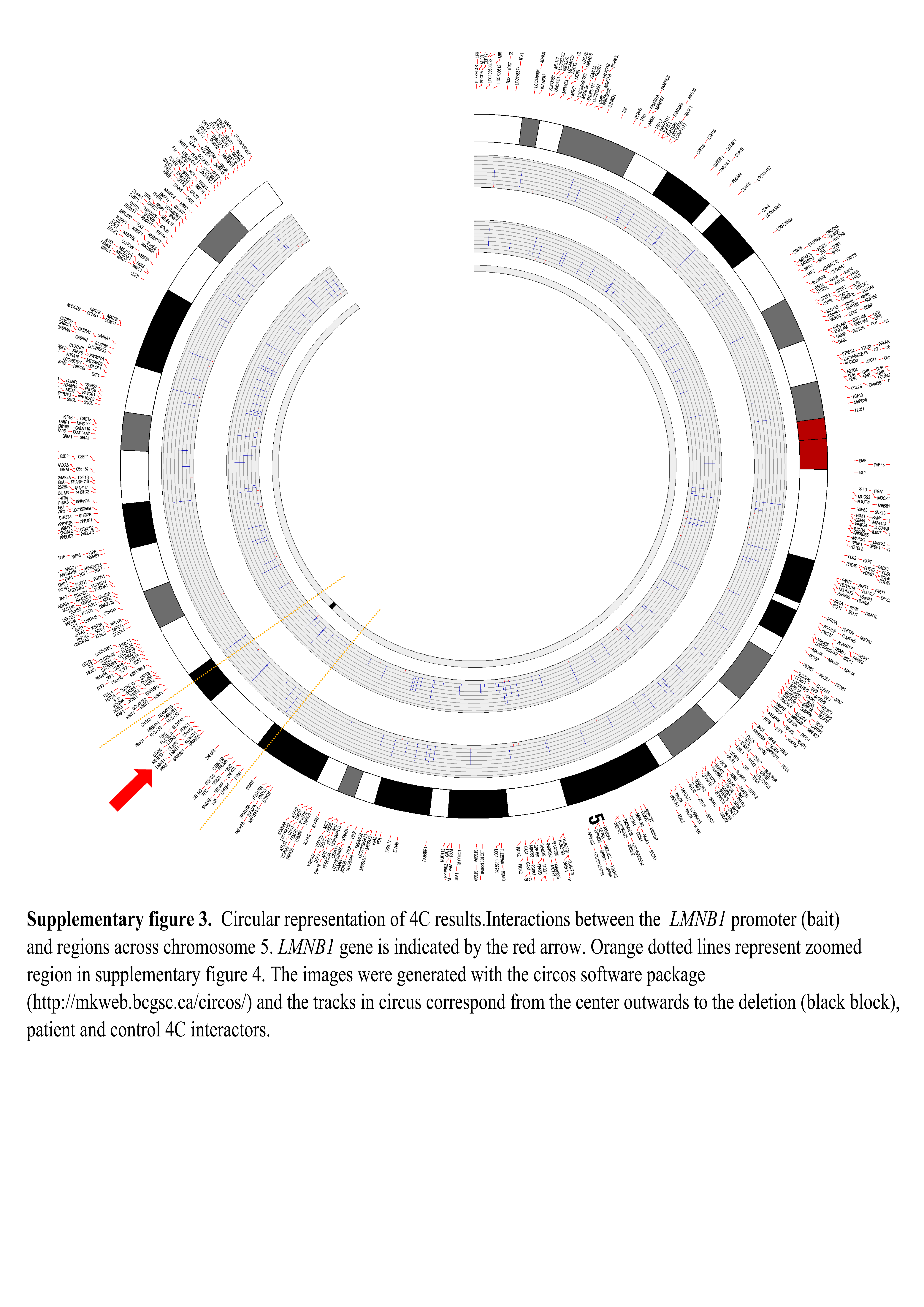

Supplement: Supplementary Data [file supp_ddv065_ddv065supp_fig3.png]

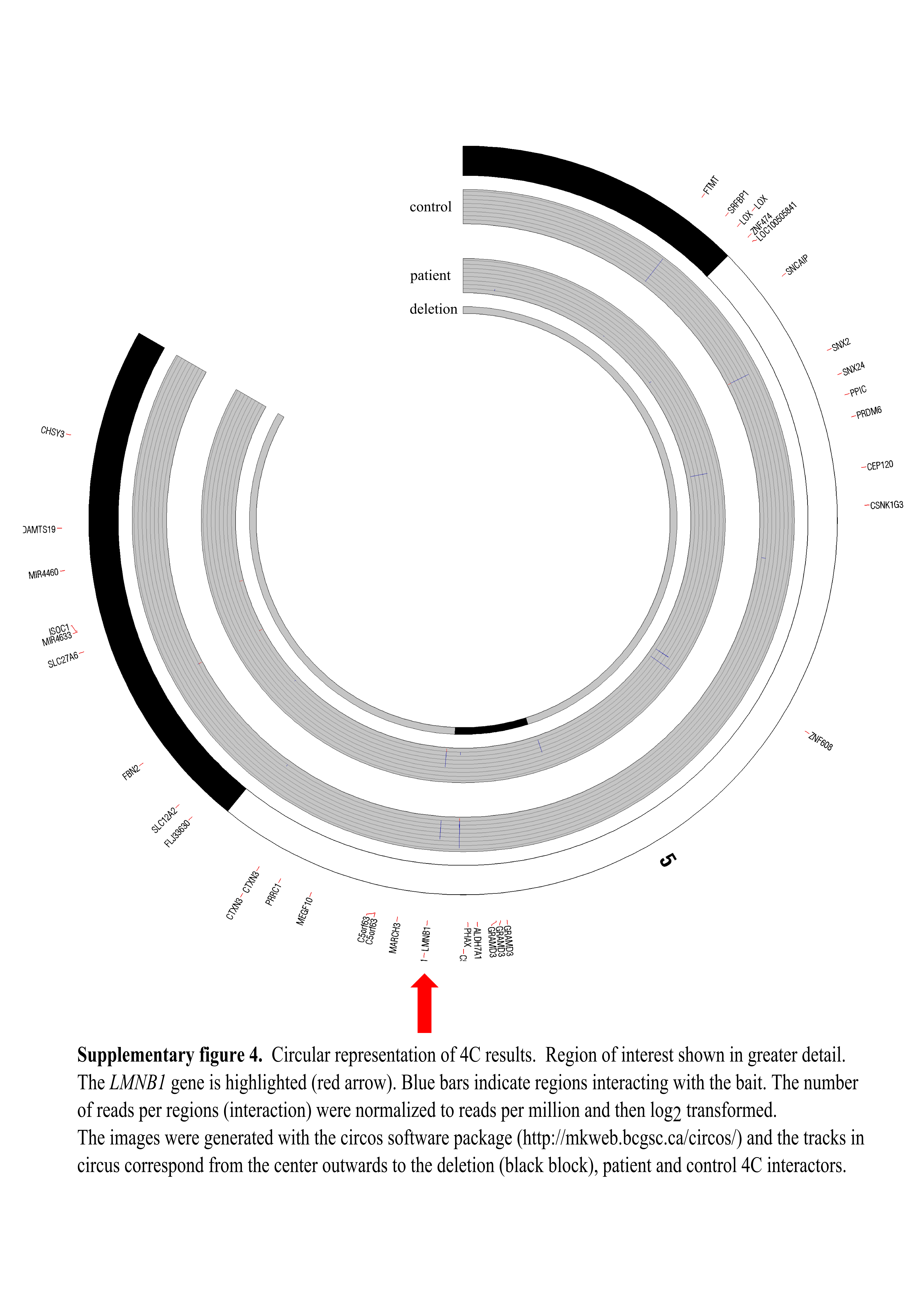

Supplement: Supplementary Data [file supp_ddv065_ddv065supp_fig4.png]

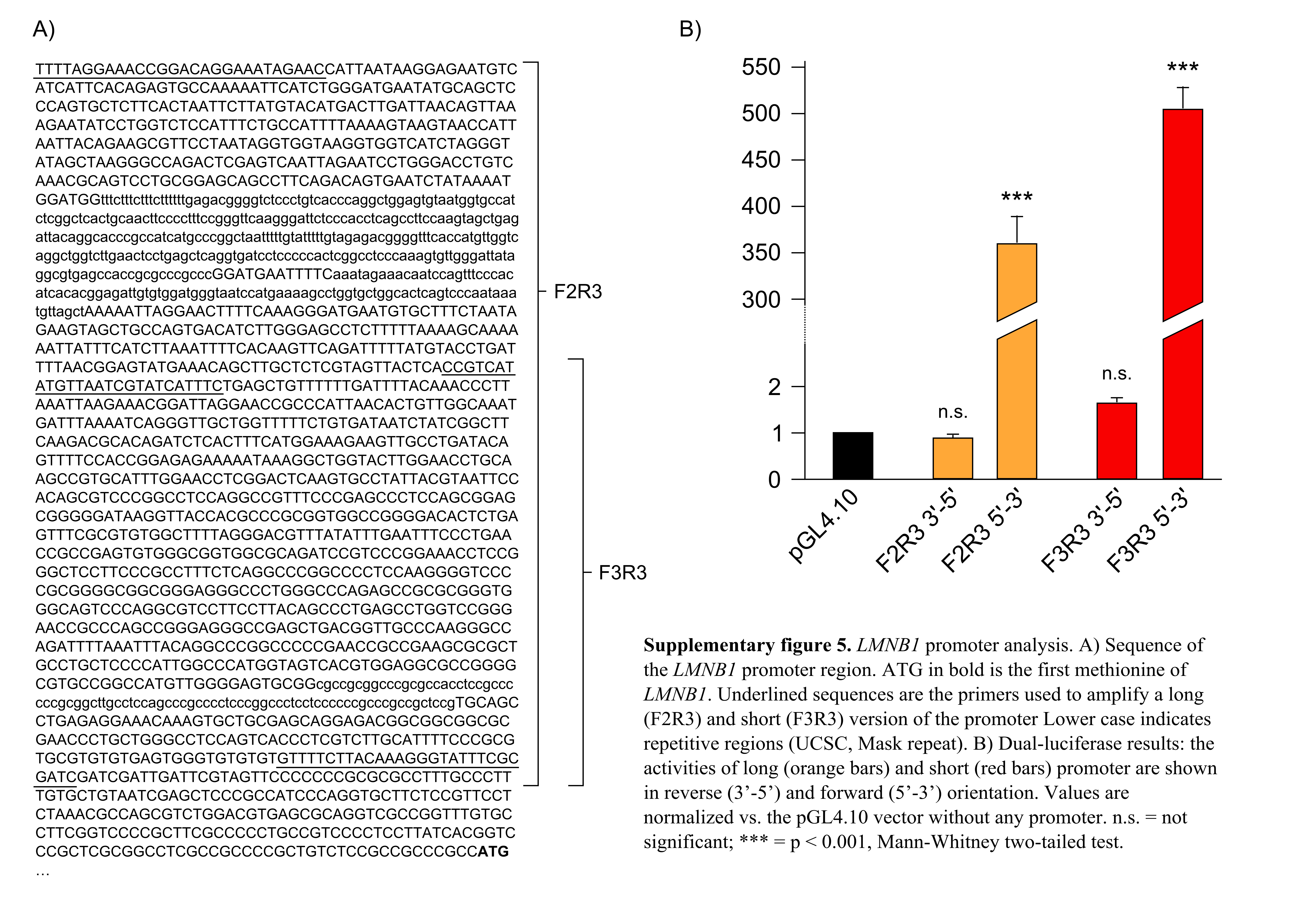

Supplement: Supplementary Data [file supp_ddv065_ddv065supp_fig5.png]

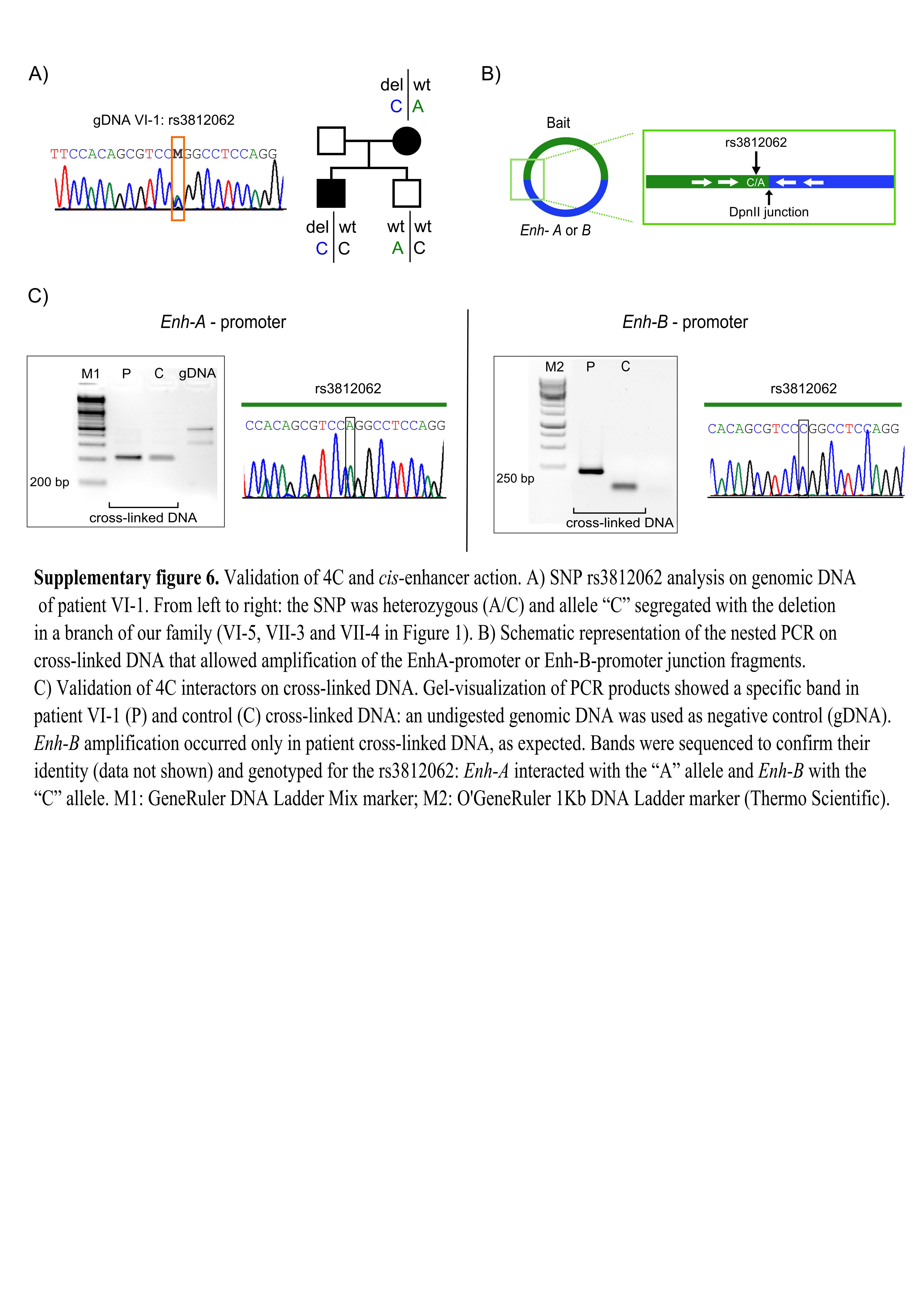

Supplement: Supplementary Data [file supp_ddv065_ddv065supp_fig6.png]
